# The chloroplast genome of *Lappula myosotis V. Wolf,* a medicinal species

**DOI:** 10.1080/23802359.2022.2158692

**Published:** 2023-01-01

**Authors:** Song Yan, Tianhao Wang, Zhen Wang, Weichao Ren, Chi Liu, Wei Ma, Shang Dong

**Affiliations:** a Pharmacy College, Heilongjiang University of Chinese Medicine, Harbin, China; b College of Mechanical and Electrical Engineering, Northeast Forestry University, Harbin, China; c Faculty of Electrical Engineering and Information Technology, Technical University of Chemnitz, Chemnitz, Germany; d Yichun Branch of Heilongjiang Academy of Forestry, Yichun, China

**Keywords:** *Lappula myosotis*, complete chloroplast genome, phylogeny, Boraginaceae

## Abstract

*Lappula myosotis V. Wolf 1776* is an annual or biennial plant with important medicinal value. In the present study, we report the complete chloroplast genome data of *L. myosotis*, which has a length of 146,668 bp, including a small single-copy (SSC) region of 17,059 bp, a large single-copy (LSC) region of 79,691 bp, and a pair of inverted repeats (IRs) of 24,959 bp. A total of 127 genes encoding tRNA and rRNA were annotated. The total CG content of the chloroplast genome was 37.7%. The maximum-likelihood (ML) phylogenetic tree strongly supported that *L. myosotis* is closely related to *Trigonotis peduncularis*. The complete chloroplast genome of *L. myosotis* provides useful information on the evolution and phylogenetic relationship among Boraginaceae plants.

## Introduction

*Lappula myosotis V. Wolf* 1776 is an annual or biennial plant of the genus *Lappula* in the family Boraginaceae. *L. myosotis* has important medicinal value and can be anti-inflammatory and insecticidal (Zhang et al. 2005). *L. myosotis* grows in grassland, hillside grassland, etc. The plant species were distributed in North China, Northwest China, and Western Inner Mongolia as well as central and Eastern Europe, North America, Afghanistan, and Pakistan (Wang et al. 1986). To better understand the genomic structure of *L. myosotis* and its phylogenetic position in Boraginaceae, we sequenced the complete chloroplast genome of *L. myosotis* and compared it with its close relatives.

## Materials

**Figure 1.**
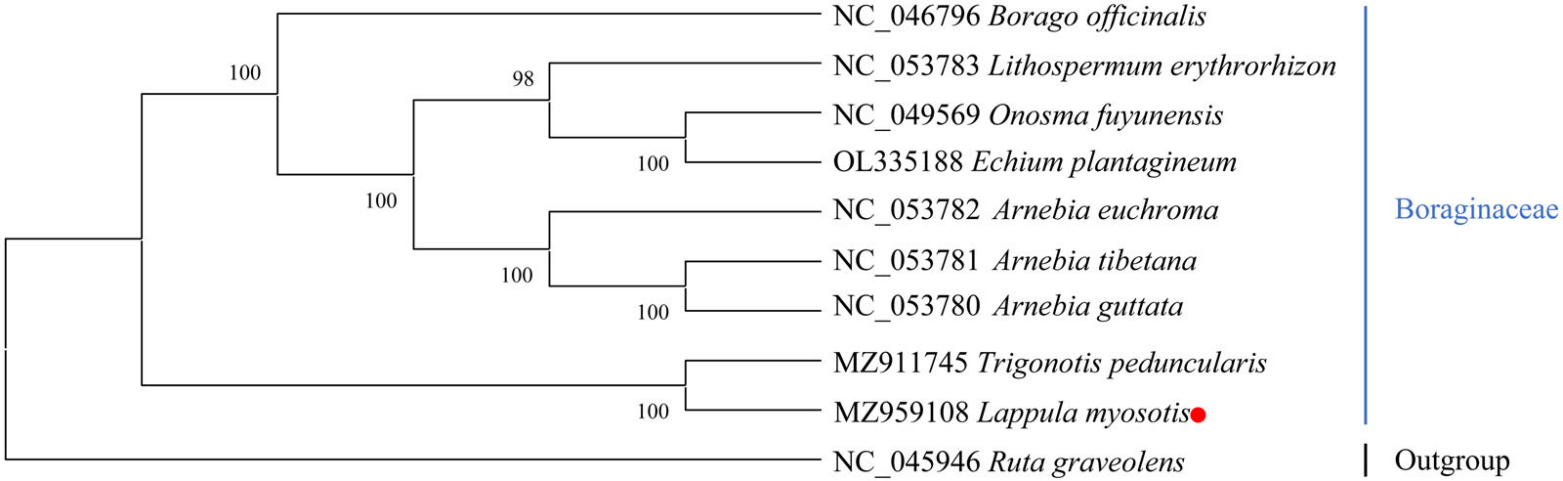
Reference image of *L. myosotis.*

We collected samples of *L. myosotis* in Heilongjiang Province, China, in June 2021 (N 44°60′16″, E 129°75′69″) (Figure 1). Voucher specimens were deposited in the Herbarium of Heilongjiang University of Chinese Medicine (registration number: MDJ20210607001) (Dr. Yan, zleztme@163.com). DNA samples were stored in the Molecular Laboratory of Heilongjiang University of Chinese Medicine (Harbin, China).

## Methods

DNA was extracted from fresh leaves of *L. myosotis* by CTAB (Hamad 2011). Genomic DNA was sequenced using an Illumina HiSeq (Benagen Co., Wuhan, China) with paired-end (PE) sequencing. The chloroplast genome of *Lithospermum erythrorhizon* (NC053783) served as our reference genome. Sequencing raw data quality was assessed using FastQC v0.11.7 software (de Sena Brandine and Smith 2019). GetOrganelle V1.7.5 (Daniell et al. 2016) software was used to splice the chloroplast genome, and GeSeq (Tillich et al. 2017) software was used for functional annotation. Quantities of rRNA and tRNA were checked using the tRNAscanSE tool (Chan et al. 2021). Finally, RAxML v8.2.12 (Stamatakis 2014) was used to construct a maximum-likelihood (ML) tree, and bootstrap values were based on 1000 replicates. The complete chloroplast genome sequence of *L. myosotis* was submitted to GenBank with accession number MZ959108.

## Results

**Figure 2.**
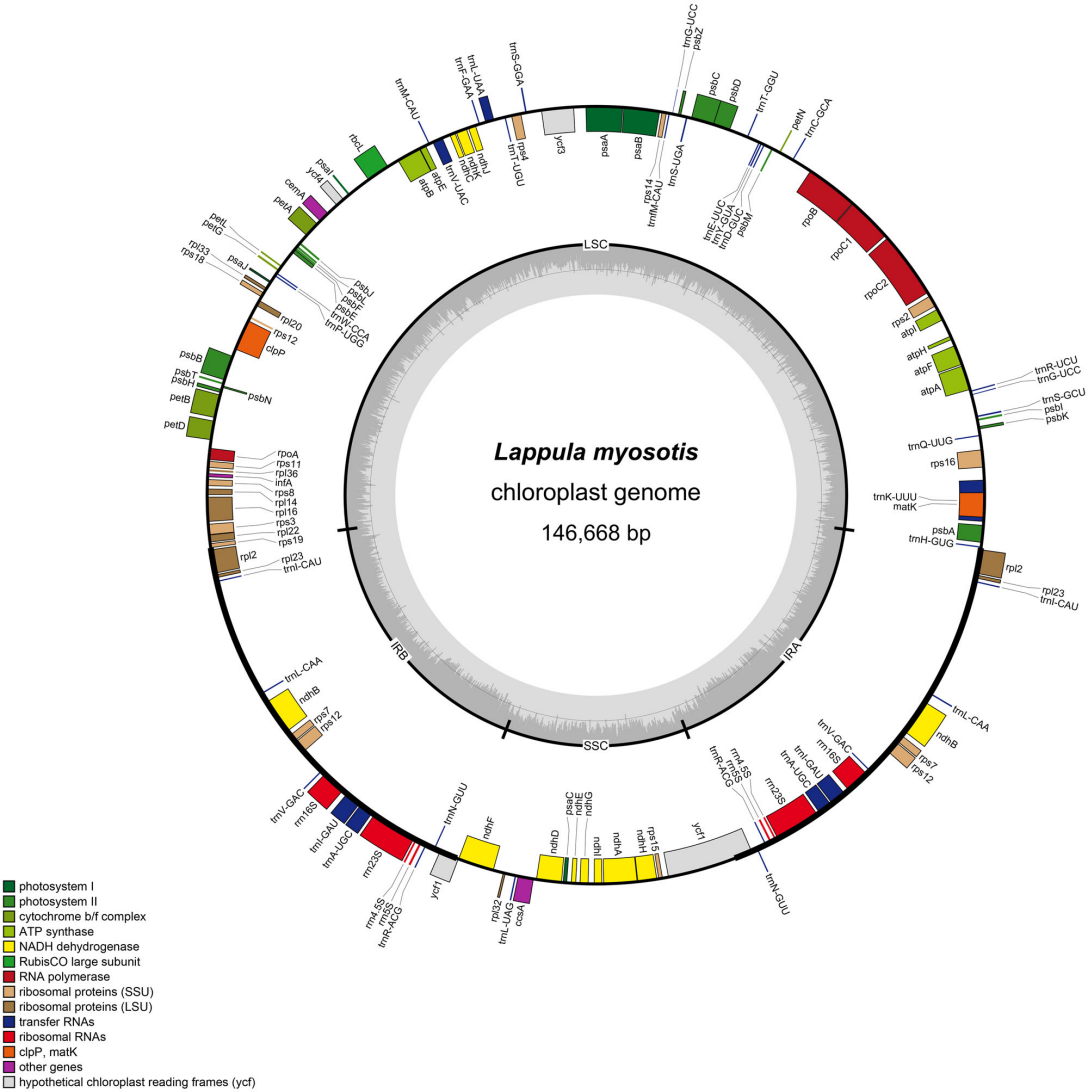
Ring map of the chloroplast genome of *L. myosotis.*

The chloroplast genome length of *L. myosotis* is 146,668 bp, which includes a large single-copy (LSC) region of 79,691 bp, a small single-copy (SSC) region of 17,059 bp and a pair of inverted repeats (IRs) of 24,959 bp (Figure 2). The total nucleotide composition was 30.7% A, 31.5% T, 19.2% C, and 18.5% G. A total of 127 genes were annotated, including 83 protein coding genes, 36 tRNAs, and eight rRNAs.

**Figure 3.**
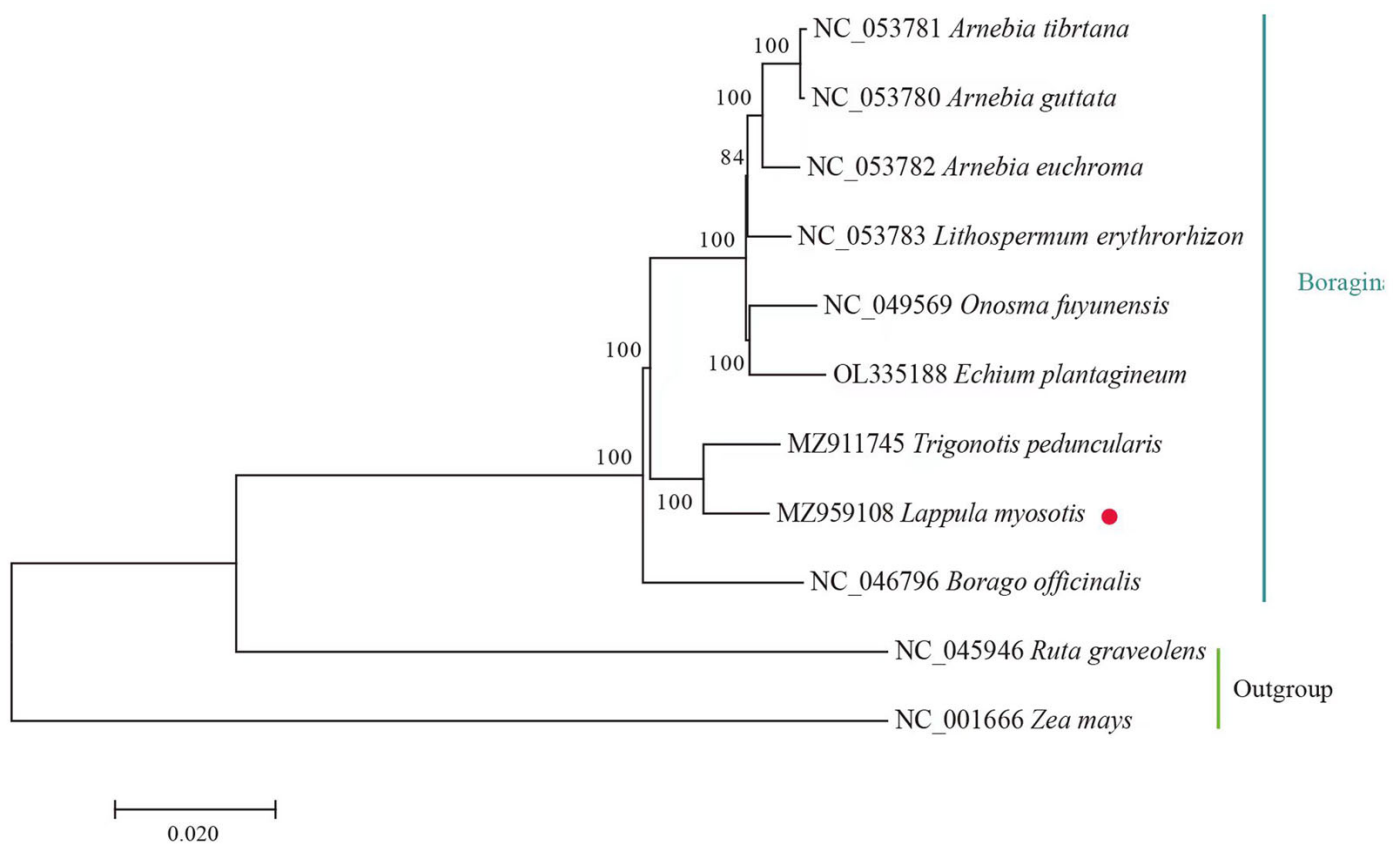
Phylogenetic tree reconstruction of 11 samples using maximum likelihood based on the complete chloroplast genome. The numbers on branches are bootstrap support values. Sequence data were obtained from the NCBI database.

To validate the phylogenetic position of *L. myosotis*, eight species from Boraginaceae and two species from Rutaceae (*Ruta graveolens*) and Poales (*Zea mays*) as an outgroup were used to construct a ML phylogenetic tree with 1000 bootstrap replications (Best-fit model: GTR + G) (Figure 3). The following sequences were used: *Arnebia tibetana* (NC_053781), *Arnebia guttata* (NC_053780), *Arnebia euchroma* (NC_053782), *Lithospermum erythrorhizon* (NC_053783), *Onosma fuyunensis* (NC_049569) (Yi et al. 2021), *Echium plantagineum* (OL335188) (Inês et al. 2022), *Trigonotis peduncularis* (MZ911745), *Borago officinalis* (NC_046796) (Zhen and Zhang 2019), and Zea mays (NC_001666) (Maier et al. 1995). The sequences were aligned using MAFFT (version: 7.313). Phylogenetic analysis showed that *L. myosotis* is most closely associated with *Trigonotis peduncularis*.

## Discussion and conclusions

The complete chloroplast genome of *L. myosotis* is the first report of a member of Lappula, which fills the gap in genome-related information. Provide data support for the subsequent classification of Boraginaceae.

## Data Availability

The genome sequence data that support the findings of this study are openly available in GenBank of NCBI at https://www.ncbi.nlm.nih.gov/ under the accession no. MZ959108. The associated BioProject, SRA, and Bio-Sample numbers are PRJNA758790, SRR15666708, and SAMN21035694, respectively.
